# Identifying research priorities for psychosocial support programs in humanitarian settings

**DOI:** 10.1017/gmh.2019.19

**Published:** 2019-10-07

**Authors:** C. Lee, A. J. Nguyen, E. Haroz, W. Tol, Y. Aules, P. Bolton

**Affiliations:** 1 Department of International Health, Johns Hopkins School of Public Health, Baltimore, MD, USA; 2 Department of International Health, Center for Refugee and Disaster Response, Johns Hopkins School of Public Health, Baltimore, MD, USA; 3 Department of Mental Health, Johns Hopkins School of Public Health, Baltimore MD, USA; 4 Department of Human Services, Curry School of Education, University of Virginia, 405 Emmet St S., Charlottesville, VA, USA; 5 Independent Contractor with the Applied Mental Health Research Group, Johns Hopkins School of Public Health, Baltimore, MD, USA

**Keywords:** Humanitarian, psychosocial

## Abstract

**Background.:**

Given the range and reach of psychosocial support (PSS) interventions in humanitarian settings, within the continuum of mental health and psychosocial support services, evaluation of their impact is critical. Understanding stakeholders' perspectives on which PSS interventions of unknown effectiveness warrant rigorous evaluation is essential to identify research priorities. This project aimed to facilitate a process with stakeholders to reach consensus on PSS interventions that are of high priority for further research based on existing evidence and stakeholders' opinions.

**Methods.:**

Interviews with 109 stakeholders working on PSS programming in humanitarian settings served as the foundation for two in-person regional meetings and four webinars. Nominal Group Technique (NGT) was used to develop a priority PSS program list. The top five priorities from each meeting were combined for a final online survey distributed globally.

**Results.:**

Seventy participants across six meetings contributed to the prioritization process. Eighty-seven individuals completed the final online survey. ‘Community based PSS’ was the top-ranked research priority, followed by PSS integrated into basic services, providing PSS to caregivers to improve child wellbeing, PSS-focused gender-based violence programming, and classroom-based PSS interventions.

**Conclusions.:**

NGT and online surveys were effective methods to engage stakeholders in a priority setting exercise to development a research agenda. Information from this stage of the project will be combined with findings from a concurrent systematic review to form the base of a second phase of work, which will include the development and implementation of a research strategy to strengthen the evidence base for those prioritized interventions.

## Background

Worldwide, over 134 million people are in need of humanitarian assistance due to conflicts and disasters, with an estimated cost of over $25 billion US dollars (UNOCHA, 2018). Many of these circumstances bring a heavy toll on mental health (MH) and psychosocial wellbeing, with impacts on the individual, family, and community at large. Given this impact, mental health and psychosocial support (MHPSS) programming is increasingly considered a core component of humanitarian response requiring multi-sectoral collaboration and a tiered framework of intervention (IASC, 2017). Although operating on a continuum, MH programming focuses on the mental disorder treatment model and psychosocial support (PSS) emphasizes prevention of disorder and promotion of wellbeing through reducing risk factors and strengthening resilience and protective factors. Although there is a growing consensus regarding the need to provide MHPSS interventions and supports, evidence to support the effectiveness of any PSS intervention, compared to the evidence that exists to support the effectiveness of MH interventions, has often been lacking (Tol *et al*. 2011*a*). Recognition of the need for rigorous research to support the MHPSS field led to development of a consensus-based MHPSS research agenda (Tol *et al*. 2011*a*, *b*), which has helped drive the field over the past decade. In addition, an earlier consensus-based statement of humanitarian MH workers at the annual Humanitarian Action Summit agreed that providing PSS programs without strong evidence for their effectiveness constitutes questionable ethical practice (Allden *et al*. 2009). There appears to be some consensus amongst international actors regarding the urgent need for rigorous evidence for PSS interventions: the need for strengthening evidence for PSS interventions was one of the main conclusions from a recent inter-agency meeting on MHPSS interventions for children affected by armed conflict (UNICEF, 2015).

In settings of adversity, PSS programs comprise a very large proportion of humanitarian programming directed at MH and wellbeing. Despite being on the MHPSS continuum, the emphasis of PSS programs is conceptually different from that of the mental disorder treatment model, resulting in a much larger collection of potential intervention approaches, targets, and outcomes than mental disorder treatment programs. This breadth presents a challenge in the field, as well as a lack of clarity around the boundaries of what is meant by ‘psychosocial’. An example of this is a series of impact evaluations of the widely-implemented child-friendly spaces (CFS) program (Metzler *et al*. 2015). Although generally perceived to be instrumental not only for psychosocial wellbeing but also for related protection and community capacity outcomes, evaluations showed that often the impacts of CFS programming are small, related to quality and fit with local context. One of the lessons learned from this work is that a safe space on its own is not necessarily a psychosocial program; rather, careful attention to promotive activities and relationships are likely necessary to promote psychosocial wellbeing. Likewise, whereas there is a perception that psychosocial considerations are relevant in a variety of basic services (e.g.: basic services with respect for dignity, considering safety and wellbeing in camp planning, etc.), these programs are typically evaluated in terms of more concrete outcomes, with lack of attention to evaluating potential psychosocial impacts of their programs.

A recent systematic review of psychotherapeutic interventions in low- and middle-income countries generally showed that these programs now have robust evidence for their effectiveness, based on at least 27 randomized controlled trials (Singla *et al*. 2017). Furthermore, evidence suggests that the delivery of these interventions by non-specialist health workers is likely to be feasible and effective (van Ginnecken *et al*. 2013). Although this robust evidence base marks a monumental achievement in the field, research-practice gaps in humanitarian settings remain (Tol *et al*.^8,9^), such that those targeted, psychotherapeutic programs are less frequently implemented in practice and reach fewer people, whereas there is less of a unified evidence base for the more commonly implemented and broader reaching PSS interventions. It appears that whereas the field has successfully developed the tools, guidelines, methods, and expertise to experimentally evaluate more targeted, person-focused interventions for impact evaluation, there remain challenges to expanding this body of research to include the more wide-reaching, often community-focused interventions in the bottom tiers of the Inter-Agency Standing Committee (IASC) pyramid. For example, evaluating impacts of a manualized MH treatment program on one or two primary MH outcomes may lend itself to the individual randomized control trial model that is often considered the gold standard in evidence-based medicine. Community-focused programming, on the other hand, may consist of multiple interventions or intervention components, in which it is unclear whether, and the extent to which, individuals were exposed to or engaged in one or many elements or activities, what target outcomes may be expected, and whether these outcomes should be measured at an individual or community level. While non-randomized controlled trial (RCT) study designs exist to evaluate community-level interventions, some form of counterfactual design is needed in humanitarian setting because of the unstable and changing circumstances that affect program outcomes and therefore obscure program effects. These effects can be large as evidenced by the large improvements in control groups in humanitarian settings which may be similar or even outweigh benefits seen in intervention conditions.

**Fig. 1.**
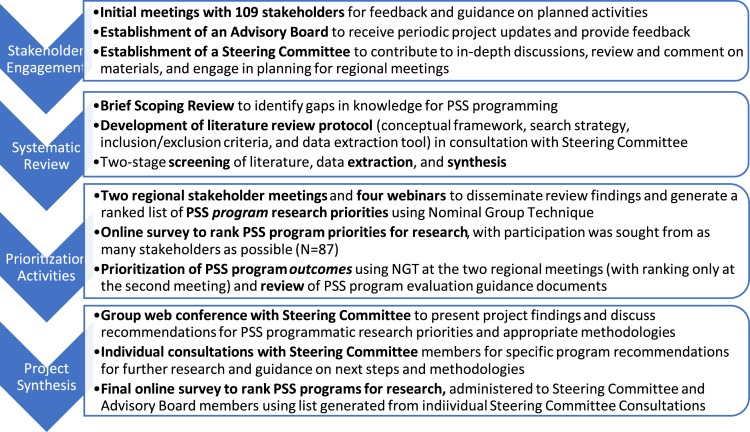
Sequence of project activities undertaken over four phases.

**Fig. 2.**
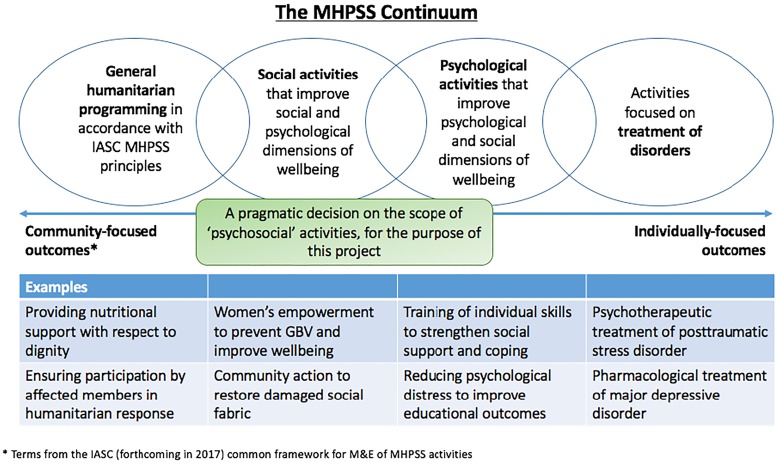
Scope of programs included in the project.

Given the range and reach of PSS interventions, evaluation of their impact is critical. With funding from the U.S. Agency for International Development (USAID) Office of U.S. Foreign Disaster Assistance (OFDA), the Applied Mental Health Research group (AMHR) at Johns Hopkins Bloomberg School of Public Health has undertaken a series of activities meant to build a research agenda for PSS interventions in humanitarian settings. Figure 1 summarizes the activities undertaken over the course of the larger project, and the purpose of each; although overlapping to an extent, these activities are organized within four sequential phases between fall 2016 and fall 2018. A core piece of this project is a large-scale systematic review (Lee *et al*. 2018) of the published and grey literature, which is the subject of a separate paper. For both the systematic review and stakeholder engagement, we focused the scope on general humanitarian programming, social activities, and psychological activities considered to be ‘interventions’, which we define as activities or groups of activities provided together to achieve a particular outcome (Fig. 2). Critical to our overall project approach is the recognition that what researchers, policy makers, and practitioners view as constituting ‘evidence’ varies, and that this may impact priorities and preferences for research and action. Parallel to the literature review this project has heavily emphasized early and ongoing stakeholder engagement, as described in this paper. Part of this engagement has been to inform and guide the literature review through a series of dissemination and feedback activities. Additionally, faced with a breadth of PSS interventions of unknown effectiveness, understanding global stakeholders' perspectives on which of these interventions warrant rigorous evaluation is essential to identify research priorities. Therefore, we further aimed to facilitate a process with stakeholders to reach consensus on PSS interventions that are of high priority for further research based on existing evidence and stakeholders' opinions.

## Methods

The current paper describes activities across several phases that involved direct stakeholder involvement, including individual consultations, of two regional meetings, four webinars, and a series of priority-setting exercises. For this project, the stakeholder group we specifically targeted was PSS program implementers, however, stakeholders involved in this project also included academics, researchers, and government officials. Therefore, the working definition of ‘stakeholder’ for this project included any individuals working directly on program implementation, research, and/or policy related to PSS programs. All activities were overseen by a 6-member Steering Committee and a 16-member Advisory Board (Table 1). Stakeholders did not receive any financial remuneration for their time and input, however, travel and daily subsistence expenses were covered by the project for those that attended the regional meetings. For all prioritization exercises, participants were briefed on the focus of the project (Fig. 2), but no specific operational definition for what interventions could be included was provided by the project.

**Table 1.** Steering Committee and Advisory Board organizations

Steering committee	Advisory board
Africa psychosocial support Institute IFRC reference centre for psychosocial support MHPSS.net Psychosocial services and training institute in Cairo TPO Uganda War child Holland	ACT Alliance Ahfad University North Sudan CARE Austria CBM International Center for Victims of Torture Church of Sweden Columbia University Independent psychologist International Medical Corps International Organization for Migration International Rescue Committee Peace in Practice Queen Margaret University Terre de Hommes UNHCR UNICEF World Vision International

### Individual stakeholder consultation

During the first 14 months of this project, AMHR sought to engage as many stakeholders as possible, drawing first from our own connections, the IASC Reference Group for Mental Health and Psychosocial Support in Emergencies (IASC RG MHPSS), and recommendations from the Steering Committee and Advisory Board. Additionally, each stakeholder we spoke with was asked to provide recommendations and introductions to others we should attempt to contact. In total, we contacted 160 stakeholders in the field of psychosocial programming. The purpose of these individual consultations was to: (1) understand stakeholder perspectives on PSS intervention evaluation research, including priorities for research and barriers to its conduct; and (2) gain stakeholder feedback and guidance around the formation and planning of our research activities to ensure they aligned with priorities in the field and were not duplicative of other efforts.

### Regional meetings

The first regional meeting was held in Bangkok, Thailand in January 2018, and the second in Kampala, Uganda in March 2018. The meetings lasted 2–3 days each and included a combined total of 27 stakeholders. AMHR selected stakeholder participants from the existing list used for individual stakeholder consultation and aimed to have representation from a variety of geographic locations and position within an organization (e.g.: field-level implementers and directors). Meeting activities included presentations and group discussion around psychosocial programming. The purpose of these meetings was threefold: (1) to continue engaging stakeholders in the overall project; (2) to share initial findings from the literature review and gain stakeholder feedback in refining review criteria and focus; and (3) to facilitate stakeholder-centered prioritization of psychosocial interventions and outcomes for further research (described below).

### Webinars

Due to limited space at the regional meetings, four additional online webinars were held between the two regional meetings to include more stakeholders in the discussions. Webinars lasted approximately 1.5 h each, included a combined total of 43 stakeholders representing 28 agencies, and were similar in purpose to the regional meetings. During the webinars, information from completed meetings and webinars was presented prior to engaging participants in an updated prioritization exercise, thereby creating an iterative process of discussion and consensus building.

### Group prioritization activities

During the two regional meetings and the interim webinars, a stakeholder-centered prioritization exercise using Nominal Group Technique (NGT; CDC, 2006) was conducted to reach consensus on priority PSS interventions for further research. NGT is a structured process similar to small-group discussion that encourages participation from all group members, can reduce domination of the discussion by particular individuals, and results in a list of prioritized items with input from all participants. The four steps for NGT are: (1) presentation of the question to the group in written form and asking each participant to write their ideas down individually, (2) presentation of individual ideas without discussion and recording of ideas in a way that is visible to the entire group, (3) discussion of ideas to allow participants to explain the items and relative importance, and (4) individual, private voting to prioritize the ideas (ranking of their top five important items) with a summary of the results visible to the entire group.

**Fig. 3.**
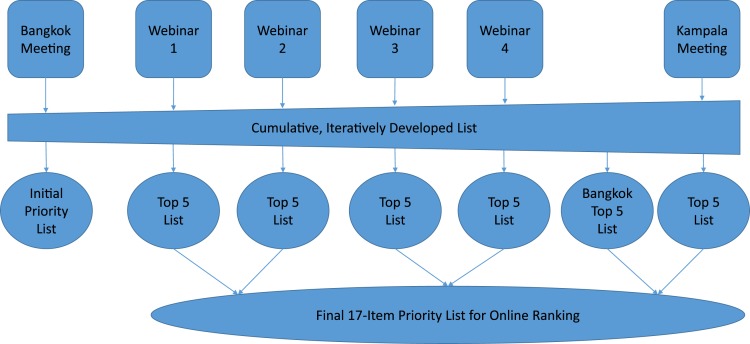
Iterative process of prioritization across all activities.

Rather than conducting the full process separately at each meeting and webinar, we adapted the approach to develop a list of programs iteratively across these meetings (Fig. 3). At the first regional meeting, following presentation of results from stakeholder consultations and the preliminary literature review findings, each attendee was asked to make a list of PSS programs to prioritize for *evaluation research*. No directives were given regarding how to prioritize programs, or what types of evaluation research was eligible. In turn, each participant presented their list along with their rationale, and the program choices were typed and projected on the screen for the participants to see in real time, resulting in a list of 45 programs. After a period of open discussion about the list, participants then completed the first round of voting in which they selected their top five PSS program priorities, resulting in a refined 33-item list. The consolidated priority list was then re-projected, for a second, similar round of voting, this time selecting their top three choices. Together, these two rounds of prioritization resulted in a consolidated, 7-item priority PSS program list that represented at least one of the top three program choices of each participant.

A similar process was used with the webinars, whereby the priority ranking process was conducted using ‘Polleverywhere’, an online polling system. Prior to the first webinar, registered participants were asked to free-list priority PSS programs for further research. These responses were then added to the initial list developed at the first regional meeting (i.e. the full list developed prior to any voting). Programs listed by more than one person (across both the regional meeting and the first webinar) were included in an online poll. At the end of the webinar, participants were asked to complete the poll, which included ranking all programs on the list in descending order of priority. Responses were tallied in real time and the prioritized list projected on the screen for webinar participants to view and discuss. The second, third and fourth webinars were conducted in the same way. Each time, pre-webinar free list responses were added to the working list, and programs listed by more than one person (across all meetings to that point) were included in the prioritization poll for that webinar.

Because of the iteratively developed list, after the fourth webinar a poll based on the updated list was sent back to participants of the first regional meeting to complete online. Additionally, during the second regional meeting, participants again generated a list of 38 PSS program priorities for research using the first three NGT steps of individual list development, sharing, and discussion. This list was then added to the cumulative list from the first regional meeting and webinars. Again, programs listed by more than one person (across all meetings to that point) were included in the online prioritization ranking, resulting in a 25-item ranking which meeting participants then completed.

### Final online prioritization

The above series of ranking activities resulted in six separate priority lists originating from a shared, iteratively generated list of potential PSS interventions. Following the second regional meeting, an online survey using Qualtrics was disseminated to all contacts from this project, as well as shared via MPHSS.net (a global platform to connect people and organizations working in the MHPSS field), to have a final round of prioritization voting open to a wide audience. For this survey, a list was generated that consisted of the top five PSS interventions from each priority list, meaning a total of potentially 30 interventions could have been listed. However, because some interventions were consistently prioritized by multiple groups, a total of 17 unique prioritized interventions were included. Respondents were asked to rank the priority interventions for further research. In addition, information was gathered on which sector(s) and geographic location(s) the respondent worked in, as well as whether the individual's organization currently implemented or had plans to implement any of these 19 interventions, and in what location and context.

## Results

### Individual stakeholder consultations

Of the 160 stakeholders we contacted, we completed individual phone consultations with 109; 77 were female and 32 were male (36 individuals did not respond to us or referred us to someone else and 15 individuals did respond to us but could not find a time to set up a meeting). Thirty-nine of these individuals worked at a global level, 44 focused in the Middle East, 16 in Africa (particularly Eastern Africa), five in Latin America, and five in Southeast Asia and the Pacific. Stakeholders represented a variety of types of organizations; 55 individuals from INGOs, 17 individuals from nongovernmental organizations (NGOs), 19 individuals worked at research centers, institutes and universities, six were independent MHPSS consultants, four worked in other type or organizations such as civil societies or social enterprises, three worked in faith-based organizations and five people worked for government bodies from different countries. The PSS programs most highly ranked for research included community-based programs, multi-sectorial programs, early childhood development programs, child friendly spaces, and family strengthening and parent skill programs (Table 2).

**Table 2.** Individual stakeholder priority PSS programs for research

Topic heading	Number of respondents
Community-based programs	21
Multi-sectorial programs	17
Early childhood development programs	17
Child-friendly spaces	16
Family strengthening and parent skills programs	15
Adolescents interventions	14
Psychological first aid (PFA)	4
GBV programs	4
Intervention in on-going crisis contexts (as opposed to post-crisis contexts)	4
Programs focused on service providers	4
Peer-to-peer support programs	3
Life skills programs	3
Disability inclusion programs	3
Programs that target men	3
mhGAP program	2
Programs with a spiritual element	2
Clinical informed activities	2

### Prioritization activities

Twelve individuals from 12 organizations attended the first regional meeting; six were female and six were male. Of these 12 individuals, six were from NGOs, three from international NGOs (INGOs), two from universities, and one from a network. In addition, the area of focus for two were in the Middle East, one in Africa, nine in Southeast Asia and the Pacific, and one in Europe. Fifteen individuals from 13 organizations attended the second regional meeting; nine were female and four were male. Of these 15 individuals, 11 were from INGOs, two from NGOs, one from a university, and one from a research institute. Eleven of these participants focused their work in Africa, two in the Middle East, and two globally. Forty-three stakeholders participated in the webinars representing 28 organizations; 29 were female and 14 were male. Seventeen participants focused their work in and joined the webinar for Middle East/North Africa/Sub Saharan Africa, seven for Southeast Asia/South Asia/Pacific, 11 for Eastern Europe/Global, and eight for Latin America/North America. Twenty were from INGOs, 12 from NGOs, three from universities, three worked as consultants, two from government, one worked as a clinical psychologist, and one from a network. Over the course of these two regional meetings and four webinars, a total of 71 individuals participated in prioritization activities that resulted in the six priority lists and contributed to the final Qualtrics survey that was shared more widely to a larger group of stakeholders. Among the top five most highly ranked PSS programs for research given from each round of voting during the meetings and webinars, several programs were highly prioritized across nearly all groups. For example, ‘MHPSS integrated into other sectors’ was prioritized in the top five PSS programs by four of the six groups. ‘Community based PSS’, ‘Gender based violence prevention programs’, ‘Programs for reduction of family violence’, and ‘Impact of PSS to parents/caregivers on outcomes for children’ were all ranked in the top five PSS programs by three of the six groups. Programs only prioritized in the top five for two groups were ‘classroom based interventions’, ‘staff care programs’, and ‘local/indigenous interventions and knowledge/practices’. Programs mentioned by only one groups for the top five PSS programs were ‘faith based groups and their activities’, ‘community mobilization methods in emergency settings’, ‘programs for survivors of human rights violations’, ‘programs that integrate PSS in emergency preparedness’, ‘psychological first aid’, ‘child friendly spaces’, ‘child peer-to-peer programs’, and ‘caregiver programs providing PSS’.

### Online survey

Eighty-seven individuals responded to the Qualtrics online survey. Participants primarily worked in the following sectors: 20 in health, 14 in protection, 11 in on-going crisis or conflict, 10 in gender equality/women's empowerment, and eight in education with the remaining sectors represented to a lesser degree being food security/nutrition, human rights, environmental/global climate change, water and sanitation, economic growth/trade, shelter/site planning, and other. Nineteen of the programs where these respondents worked were in South Asia, 17 in the Middle East and North Africa, 15 Sub-Saharan Africa, and 15 global. Only eight, eight, and four of programs they work to implement were in Latin America and the Caribbean, Europe and Central Asia, and East Asia and Pacific, respectively. The most highly ranked programs for further research on effectiveness by those who participated in the final prioritization were community-based PSS, MHPSS integration into other sectors, PSS interventions for parents/caregivers with outcomes for children, PSS in gender-based violence prevention programs, and classroom-based interventions (Table 3).

**Table 3.** Results from the Qualitrics online survey prioritization

Ranking (Most to least votes)	Program/Area
1	Community-based PSS
2	MHPSS integrated into other sectors – specifically first layer of pyramid
3	Impact of psychosocial support to parent/caregivers on outcomes for children
4	Gender-based violence prevention programs
5	Classroom-based interventions
6	Suicide prevention *and* psychological first aid (Tie vote)
7	Staff care programs
8	Community mobilization methods in emergency
9	Programs that integrate PSS into emergency preparedness
10	Programs for survivors of human rights violations – mass graves, torture, disappearances, former child soldiers
11	Healing practices related to community's spirituality and environment and Nature and psycho-spiritual healing
12	Caregiver programs providing PSS
13	Faith-based groups and their activities, particularly in emergencies, faith leaders providing PSS
14	Local/indigenous interventions and knowledge/practices
15	Child-friendly spaces
16	Programs for reduction of family violence (IPV and child-related)

## Conclusions

As a complement to the parallel literature review, input from stakeholders in the field of PSS programming is important to understand more about their prioritization of where the gaps are for evaluation research on PSS. The literature review findings were used to inform stakeholders about available studies on the effectiveness of PSS interventions to guide them in setting priorities, however, stakeholder input was a key part of the prioritization process. Input from stakeholders allows us to identify gaps and priorities that might not be found in the literature (particularly given the gap between what has traditionally been the focus of researchers, and the focus of practitioners), as well as to be able to compare stakeholder views to the literature review. In addition, engaging and respecting the stakeholders' views on research priorities is useful for gaining buy-in and collaboration on future research in line with issues considered to be priorities. Despite the time involved to contribute to this process, stakeholders expressed appreciation for being involved. Although time consuming to conduct the large number of individual consultations, this effort allowed for diverse and detailed input from a range of stakeholders. We found that the individual discussions, compared to webinars, provided more detailed information and allowed the team to gather additional contacts for further discussions. The stakeholders we spoke with were highly supportive of further research efforts, but discussed barriers to conduct this research, particularly lack of tools and organizational research capacity amongst humanitarian agencies. In addition, given the breadth of PSS programming, stakeholders provided a wide range of PSS programs for the prioritization exercise and often had difficulty identifying specific interventions given the focused nature of their own individual work.

Use of the Nominal Group Technique across all regional meetings and webinars served as a useful, iterative process to reach consensus on prioritized PSS areas for further research and captured input from a wide range of stakeholders. Stakeholders expressed that they appreciated the chance to give input into this phase of the project and found the NGT process both in-person and online to be an accessible and acceptable way for them to prioritize their ideas. In addition, responses were calculated in real time during webinars to show the final list to participants and solicit any further feedback. While use of NGT online is not a new method, for this study it allowed us to optimize feedback in a way that would lead to setting of the research priorities. This process can serve as an example for future exercises to involve a wide range of stakeholders in prioritization and ranking related to MHPSS and other fields. We found that use of the online voting (Qualtrics and Polleverywhere) were extremely valuable tools that allowed for timely and efficient collection of information. Although the webinars did contribute to achieving our goals, in future use of these methods we feel it would be useful to include plans and budget for more in person regional meetings, as well as making some changes to the webinars. The changes for the webinars include holding more online sessions with fewer participants and including video conferencing – both of which might increase participation and discussion.

PSS program implementers identified a strong preference to focus future research efforts on community-focused programs. ‘Community based PSS’ was the top-ranked programmatic research priority, followed by PSS integrated into basic services, providing PSS to caregivers to improve child wellbeing, PSS-focused gender-based violence programming, and classroom-based PSS interventions. This view is echoed in recent international meetings on MHPSS (*Growing Up in Conflict: The Impact on Children*'*s Mental Health and Psychosocial Well-being* (UNICEF, 2015); *Wilton Park: Healing the Invisible Wounds of War* (Save the Children, 2018); *MHPSS Expert Meeting: Addressing Needs, Scaling-up and Increasing Long-term Structural MHPSS Interventions in Protracted and Post-Conflict Settings* (UNICEF, 2018); *DFID Interagency Roundtable: Children & Families in Armed Conflict*, October 2018). Advocacy messages across these meetings reflect this emphasis on community-focused approaches to promote social cohesion and peacebuilding; strengthen multi-layered and inter-sectoral approaches to promote wellbeing; build resilience and improve social ecology; attain rigor in research on community-based PSS; achieve quality and scale of PSS interventions; and fund innovative community-based MHPSS programs and research.

Findings show there is a high level of interest in research on effectiveness of PSS interventions. Although there are different perceptions of what constitutes evidence of impact and little evidence is available for PSS interventions, as was found in the literature review associated with this project, the focus should be on building in evaluations of PSS interventions.

One major finding from this study is that the current body of evidence of impact of PSS interventions is poor. A great deal of resources continues to be put toward interventions without a strong existing evidence base and for which there are no embedded activities to measure impact. Even routine impact evaluation that forms part of standard program monitoring and evaluation is not commonly done. When done, these impact evaluations tend to be weak and there is a lack of focus on replicating and evaluating the same programs to build the evidence base. A suggestion from this study is for donors and agencies to institute a policy that PSS interventions should either be proven effective prior to implementation or be implemented as part of an impact study to make the best use of resources to contribute to progress in improving the effectiveness of PSS interventions in humanitarian settings, while focusing on replication of evaluations on the same program in different settings. Programs that cannot demonstrate their impact should no longer be accepted.

Stakeholder input from this prioritization exercise will be used alongside findings from the literature review to develop proposals for phase 2 of the project. Phase 2 proposal will include development of new methods and tools to support rigorous evaluation of community focused programs. These methods and tools will be tested in several field-based studies of highly prioritized community-focused PSS programs.

### Strengths and limitations

A strength of this approach was the iterative process that allowed for large amounts of input from a variety of stakeholders. In addition, the iterative process for prioritization allowed for all responses to be incorporated and built upon going forward, rather than only gathering information from individual participant groups (e.g. regional meetings and webinar).

However, limitations include that our process may not have captured input from many field-based stakeholders and responses were limited to English language. In addition, geographic representation from South and East Asia, as well as South America, was lacking despite efforts to identify stakeholders in these areas during the process. Also, donors were not specifically recruited as stakeholders to attend or give input during the activities. However, the final prioritization online survey was open to all globally through mhpss.net. For future, similar efforts, we suggest that donors who support PSS programs be specifically sought out to include as stakeholders in the process while coding their responses separately in order to compare to the responses of stakeholders who are program implementers and researchers. Finally, although participants were provided an overview of the scope of this project as it relates to how PSS was differentiated from MH, respondents to our prioritization exercises may not have used the same distinction when providing their input.

In conclusion, this comprehensive stakeholder presentation identified enthusiasm amongst stakeholders to focus future evaluation research on PSS interventions in humanitarian settings, especially with a focus on community-based PSS.
